# Biostimulant Effects of Seed-Applied Sedaxane Fungicide: Morphological and Physiological Changes in Maize Seedlings

**DOI:** 10.3389/fpls.2017.02072

**Published:** 2017-12-06

**Authors:** Cristian Dal Cortivo, Giovanni Battista Conselvan, Paolo Carletti, Giuseppe Barion, Luca Sella, Teofilo Vamerali

**Affiliations:** ^1^Department of Agronomy, Food, Natural Resources, Animals and the Environment, University of Padua, Padua, Italy; ^2^Department of Land, Environment, Agriculture and Forestry, University of Padua, Padua, Italy

**Keywords:** biostimulant, hormone-like activity, nitrogen metabolism, phenolic acids, root branching, succinate dehydrogenase inhibitor (SDHI)

## Abstract

Most crops are routinely protected against seed-born and soil-borne fungal pathogens through seed-applied fungicides. The recently released succinate dehydrogenase inhibitor (SDHI), sedaxane^®^, is a broad-spectrum fungicide, used particularly to control *Rhizoctonia* spp., but also has documented growth-enhancement effects on wheat. This study investigates the potential biostimulant effects of sedaxane and related physiological changes in disease-free maize seedlings (3-leaf stage) at increasing application doses (25, 75 and 150 μg a.i. seed^-1^) under controlled sterilized conditions. We show sedaxane to have significant auxin-like and gibberellin-like effects, which effect marked morphological and physiological changes according to an approximate saturation dose-response model. Maximum benefits were attained at the intermediate dose, which significantly increased root length (+60%), area (+45%) and forks (+51%), and reduced root diameter as compared to untreated controls. Sedaxane enhanced leaf and root glutamine synthetase (GS) activity resulting in greater protein accumulation, particularly in the above-ground compartment, while glutamate synthase (GOGAT) activity remained almost unchanged. Sedaxane also improved leaf phenylalanine ammonia-lyase (PAL) activity, which may be responsible for the increase in shoot antioxidant activity (phenolic acids), mainly represented by *p-*coumaric and caffeic acids. We conclude that, in addition to its protective effect, sedaxane can facilitate root establishment and intensify nitrogen and phenylpropanoid metabolism in young maize plants, and may be beneficial in overcoming biotic and abiotic stresses in early growth stages.

## Introduction

In intensive agriculture, seed coating is a technique of applying several compounds, such as pesticides, fertilizers and biostimulant substances, to the seed surface so they can start to act on the seedlings during germination and/or at the seed-soil interface immediately after sowing (Ehsanfat and Modarres-Sanavy, 2005).

Protecting field crop plants from soil- and seed-borne pathogens during germination and in early growth stages is crucial to ensure safe and fast establishment (Mathre et al., 2001). Fungicides are chemical and biological compounds that kill pathogenic fungi or inhibit fungal spore germination (McGrath, 2004), and, together with insecticides, are the molecules most frequently used in the seed coatings of many crops.

A fungicidal seed treatment is commonly composed of a trace quantity of fungicide evenly distributed among the seeds along with the adhesive substances needed to bind them to the seed surface (Sharma et al., 2015). Modern seed dressing fungicide formulations are often a mixture of several active ingredients with different modes of action (systemic and contact), which broadens the spectrum of control to include a wide range of pathogens and reduces the likelihood of resistance onset (Kitchen et al., 2016). Common fungicide combinations for cereals are triticonazole + prochloraz (Krzyzinska et al., 2005; Vermeulen et al., 2017), both sterol-inhibiting fungicides, and fludioxonil + metalaxyl-M (Mondal, 2004), the former a non-systemic phenylpyrrole, which inhibits transport-associated phosphorylation of glucose, the latter an acilalanine RNA synthesis inhibitor.

Substances on the seed surface can affect germination, as they may vary considerably in the degree to which they attract or repel moisture (Scott, 1989). When applied in high concentrations, fungicides have been reported to have potential direct negative effects on seed germination, rootlet growth, and emergence (Minamor, 2013). In many cases, the effects of seed-applied fungicides on plants vary according to growing conditions: under low pathogen pressure, they do not improve crop emergence and grain yield of wheat, but under high pressure from *Fusarium graminearum* they do (May et al., 2010). Environmental factors may also play a role (Cox and Cherney, 2014). Seed coating is expected to suppress arbuscular mycorrhizal fungi, hindering their colonization of roots and consequently reducing their beneficial effects on plant growth (Chiocchio et al., 2000; Channabasava et al., 2015).

In the search for highly effective active ingredients, attention is currently focused on useful secondary effects of fungicides on seedling development, regardless of genotype and growing conditions. Several fungicides have been found to have positive side-effects on plant physiology (Berdugo et al., 2012): The ubiquinol oxidase inhibitor (Qol) Strobilurin family is known to increase several morphological traits of maize, such as leaf number and area, and shoot and root biomasses (Lazo and Ascencio, 2014). Strobilurins have also been found to increase tolerance to abiotic stresses, as they can delay senescence of the photosynthetic leaf area, change the balance of the phytohormones, and increase CO_2_ assimilation in wheat (Wu and von Tiedemann, 2001; Köhle et al., 2002). The azole fungicide class also influences the physiology of treated plants by increasing the chlorophyll content in winter wheat plants, delaying leaf senescence, and protecting plants from several abiotic stresses (Fletcher et al., 2010).

Recent studies have demonstrated the influence of pyrazole-carboxamide succinate dehydrogenase inhibitors (SDHIs) on plant physiology (Ajigboye et al., 2014, 2016). These comprise a relatively new class of fungicide (since 2000), and now include various active ingredients, such as boscalid, bixafen, isopyrazam and sedaxane, which can disrupt fungal respiration causing a breakdown in energy/ATP production (Avenot and Michailides, 2010). The SDHI sedaxane (Syngenta Crop Protection, Basel, Switzerland) has recently been released for use as a treatment for local and systemic protection of cereal seeds, seedlings and roots against pathogenic fungi, both seed-borne (*Ustilago nuda, Tilletia caries, Monographella nivalis, Pyrenophora graminea*) and soil-born (*Rhizoctonia solani, R. cerealis, Gaeumannomyces graminis*, *Typhula incarnata*) (Zeun et al., 2013; Ajigboye et al., 2016). When sedaxane moves from the seed to the soil and into the plant tissues, it has been found to improve the development of the roots and lower stems of cereals (Swart, 2011). Previous research has described wheat responding positively to sedaxane in terms of greater biomass, better growth and drought resistance (Ajigboye et al., 2016). These morpho-physiological reactions are also known to be induced by biostimulants (Calvo et al., 2014), defined as substances that at low doses are able to enhance hormone biosynthesis, nutrient uptake from the soil, resistance to biotic/abiotic stresses, crop quality, and root growth (Kauffman et al., 2007).

Given all this, the present study aimed to investigate the potential biostimulant activity of seed-applied sedaxane on maize plants, and the possible physiological mechanisms underlying the morphological changes. To this end, we: (i) carried out a bioassay (Audus test) to determine the biostimulant activity of sedaxane, (ii) measured the morphological variations in pot-cultivated, disease-free maize plants at increasing fungicide doses, and (iii) studied the response of the enzymes involved in nitrogen and phenylpropanoid metabolism, and the protein, sugar and total phenol contents in the leaves and roots of the same plants.

## Materials and Methods

### Characteristics of Sedaxane

In this study, we used the fungicide formulation Vibrance^®^ 500 FS, a commercial flowable concentrate for seed treatment containing 500 g sedaxane^®^ L^-1^, i.e., 43.7% w/w of AI (density 1.17 g mL^-1^; pH 6.39). Sedaxane is the ISO common name for a mixture of two *cis*-isomers, 2′-[(1RS,2RS)-1,1′-bicycloprop-2-yl]-3-(difluoromethyl)-1-methylpyrazole-4-carboxanilideand two *trans*-isomers 2′-[(1RS,2SR)-1,1′-bicycloprop-2-yl]-3-(difluoromethyl)-1-methylpyrazole-4-carboxanilide (IUPAC). Its minimum purity is 960 g kg^-1^, with ranges of 820–890 g kg^-1^ for the 2 *trans*-isomers (SYN508210 – 50:50 mixture of enantiomers), and 100–150 g kg^-1^ for the 2 *cis*-isomers (SYN508211 – 50:50 mixture of enantiomers) (EFSA, 2012).

### Pot Trial Set-Up and Plant Analysis

Plants of the maize hybrid Hydro (Syngenta, Basel, Switzerland) were grown in cylindrical PVC pots (50 cm high, 9 cm diameter, 3.1 L volume) in a greenhouse in the L. Toniolo experimental farm of the University of Padua (Legnaro, NE Italy). The pots were filled with a sterilized mixture (36 h in an oven at 120°C) of silty-loam soil collected from a field on the experimental farm (pH 8.4), and fine sand (1:1 w/w) to facilitate water drainage and root collection, to which was added a standard dose of pre-sowing fertilizer (about 100 kg N ha^-1^, 150 kg P_2_O_5_ ha^-1^ and 300 kg K_2_O ha^-1^). Maize seeds were treated with three increasing doses of sedaxane: 25, 75, and 150 μg AI seed^-1^, corresponding to label doses of 2.5, 7.5, and 15 mL of the commercial product Vibrance^®^ 500 FS (500 g AI L^-1^) in 50,000 seeds. Plants grown from treated seeds were compared with untreated controls. The experimental design was completely randomized with 6 replicates.

Three seeds per pot were sown at the end of June, and immediately after emergence plants were thinned to one per pot. At harvest, growth measurements were taken from three pots/plants, and enzymatic activity assays were carried out with a further three.

Water stress was avoided throughout the experiment by regularly watering the plants. Before plant harvest, which took place 20 days after sowing (DAS) at the 3-leaf stage, SPAD (Soil Plant Analysis Development) was measured in the last fully developed leaf with a 502 chlorophyll meter (Konica-Minolta, Hong Kong). Fresh and dry (oven-dried for 24 h at 105°C) weights were measured on three replicate samples of shoots, and roots were collected, gently washed of soil, and stored in a 15% v/v ethanol solution until morphological characterization. Root length, surface area, diameter, and number of tips and forks were measured by analysis of 1-bit 400-DPI images of the roots acquired with a flatbed scanner (Epson Expression 11000XL, Epson, Suwa, Japan) using the WinRhizo software (Regent Instruments, Ville de Québec, QC, Canada).

Three replicates were stored at -80°C until analysis, then shoot and root tissue samples were taken from them for enzymatic activity assays. Each enzymatic assay (*n* = 9) was carried out in triplicate on each plant.

A further trial was performed following the same procedure and timing of the main experiment, and using the same sand-soil mixture (1:1 w/w), but this time it was not sterilized. We took SPAD readings, and measured fresh and dry weights, and root morphological parameters of plants grown in unsterilized soil, as reported above (Supplementary Table S1).

### Bioassay to Test the Biological Activity of Sedaxane

In order to investigate the biological activity of sedaxane, we measured the reduction in root growth in the model plant watercress (*Lepidium sativum* L.) to assess auxin-like activity, and the increase in shoot length in lettuce (*Lactuca sativa L*.) to assess gibberellin-like activity (Audus, 1972).

Watercress and lettuce seeds were surface-sterilized by immersion in 8% hydrogen peroxide for 15 min. After rinsing 5 times with sterile distilled water, 20 seeds were aseptically placed on filter paper in a Petri dish. In the case of watercress, the filter paper was moistened with 1.2 mL of H_2_O (controls), or with 1.2 mL of 0.1, 1, 10 and 20 mg L^-1^ indoleacetic acid (IAA, natural auxin) (Sigma–Aldrich, St. Louis, MO, United States) to obtain the calibration curve, or with 1.2 mL of a serial dilution of the tested product Vibrance containing 500 g L^-1^ of AI sedaxane. The experimental design for lettuce was the same as for watercress, except that the sterile filter paper was moistened with 1.4 mL of the above solutions, while the calibration curve was a progression of 0.0001, 0.001, 0.01, and 0.1 mg L^-1^ gibberellic acid (GA) (Sigma–Aldrich, St. Louis, MO, United States).

Seeds were germinated in the dark at 25°C. After 48 h, the watercress seedlings were removed from the dishes and the roots measured with a digital gauge; after 72 h, the lettuce seedlings were removed and the shoots measured with a digital gauge.

A linear regression model (y = a + bx) was used to describe the dose-response relationship after logarithmic transformation of IAA, GA and the sedaxane doses, where x was the sedaxane concentration (g L^-1^) and y the root or shoot length (mm) (Conselvan et al., 2017).

### Protein Extraction and Determination

Fresh leaf and root samples, previously stored at -80°C, were ground to a homogenous powder with liquid N_2_. Proteins were extracted by homogenizing 0.5 g of root or shoot materials with 5 mL of 38 mM KH_2_PO_4_ and 62 mM K_2_HPO_4_, pH 7, at 4°C. After 2 min, the extract was filtered through three layers of muslin and centrifuged at 15,000 *g* for 20 min at 4°C. A 50-μL supernatant sample was incubated with 50 μL of Milli-Q water and 2.5 mL of 0.00117 M Bradford reagent. After 15 min, the protein concentration in the extract was determined according to Bradford (1976), using a Jasco V-530 UV/Vis spectrophotometer (Jasco Corporation, Tokyo, Japan) at 595 nm wavelength. The protein concentration was expressed as mg of protein per g of fresh root or shoot.

### Enzyme Extraction and Assay Conditions

To extract the enzymes involved in N reduction and assimilation pathways, fresh shoot and root samples were ground to a homogeneous powder with liquid N_2_. Each activity assay was carried out in triplicate and with 3 biological repetitions using specific buffers for enzyme extraction.

Glutamine synthetase (GS; EC 6.3.1.2) was extracted by homogenizing 0.6 g of root or shoot material at 4°C with 2.4 mL of a solution of 1 mM Tris(hydroxymethyl)aminomethane HCl (Tris-HCl), 25 mM KH_2_PO_4_, 10 mM L-cysteine hydrochloride monohydrate and 3% (w/v) bovine serum albumin at pH 7.8 (Baglieri et al., 2014). After 10 min, the extract was filtered through two layers of gauze and centrifuged at 15,000 *g* for 25 min at 4°C. A 200-μL sample of supernatant was incubated with 200 μL of reaction buffer (50 mM Tris-HCl, 20 mM MgSO_4_, 80 mM L-glutamate, 30 mM NH_2_OH, 24 mM ATP, pH 7.8) at 37°C for 25 min. Reaction was blocked with a stopping solution (0.5 mL of 370 mM FeCl_2_⋅6H_2_O and 670 mM HCl). Samples were centrifuged at 15,000 g for 15 min. The amount of γ-glutamyl hydroxamate in the supernatant was determined photometrically (wavelength 540 nm) against an immediately stopped parallel sample (Jezek et al., 2015). A standard curve was made using authentic γ-glutamyl hydroxamate (GHA) proportional to absorbance intensity. Enzyme activity was expressed as μmol of GHA produced per g of fresh root or leaf tissue per minute (Conselvan et al., 2017).

Glutamate synthase (GOGAT; EC 1.4.7.1) was extracted by homogenizing 0.5 g of root or shoot material with 2 mL of a solution of 100 mM Tris-HCl, pH 8.2, 10 mM MgCl_2_⋅6H_2_O, 2 mM β-mercaptoethanol, 10% (v/v) glycerol and 1 mM Na_2_EDTA. After 15 min, the extract was filtered through two layers of gauze and centrifuged at 15,000 *g* for 30 min at 4°C. The supernatant was centrifuged a second time at 15,000 *g* for 15 min at 4°C. For the enzyme assay, 100 μL of extract was added to 900 μL of reaction buffer (41.6 mM HEPES, pH 7.5, 1 mM NADH, 10 mM EDTA, 20 mM glutamine) and 300 μL (for leaf extract) or 900 μL (for root extract) of 10 mM α-ketoglutaric acid. The reaction time was 2 min for the shoot extract, and 1.5 min for the root extract at 30°C. GOGAT was assayed spectrophotometrically by monitoring NADH oxidation at wavelength 340 nm according to Avila et al. (1987). GOGAT activity was expressed as nmol NADH reduced per g of fresh root or shoot per minute.

For the phenylalanine ammonia-lyase (PAL; EC 4.3.1.5) assay, 1 g of shoot material was homogenized with 0.1 g of poly(vinylpolypyrrolidone) (PVPP) and 5 mL of 100 mM potassium phosphate buffer (pH 8.0) containing 1.4 mM β-mercaptoethanol. After 10 min, the extract was filtered through two layers of gauze and centrifuged at 15,000 *g* for 20 min at 4 °C. A 60 μL sample of supernatant was incubated with 400 μL of 100 mM Tris-HCl buffer (pH 8.8), 140 μL of 100 mM phosphate buffer and 200 μL of 40 mM phenylalanine, at 37°C for 30 min. Reaction was stopped with 200 μL 6N HCl (El-Shora, 2002). After centrifuging at 10,000 *g* for 15 min, the absorbance of the supernatant was measured at 280 nm against an immediately stopped parallel sample. A standard curve was made using authentic cinnamic acid at increasing dilutions. PAL activity was expressed as nmol cinnamic acid produced per mg of protein in the sample per minute.

### Soluble Phenol Extraction and Determination

Soluble phenolic acids were extracted by homogenizing 200 mg of leaf material with 600 mL of pure methanol. The extract was kept on ice for 30 min then centrifuged at 15,000 *g* for 30 min at 4°C. Total phenols were measured according to the procedure described by Arnaldos et al. (2001). In brief, 1 mL of 2% Na_2_CO_3_ and 75 μL of Folin-Ciocalteau reagent (Sigma–Aldrich, St. Louis, MO, United States) were added to 50 μL of the phenolic extract. After incubation in the dark for 15 min at 25°C, absorbance was measured at 725 nm. A standard curve was made using authentic gallic acid. The soluble phenol content was expressed as mg of gallic acid equivalent (GAE) per g of fresh shoot material.

Free phenolic acid concentrations were revealed on 0.1 g shoot samples treated with 5 mL 80% (v/v) acetonitrile (ACN) in 10-mL tubes for 5 min at room temperature with agitation (70 rpm). After centrifugation (5 min, 10,000 RCF), clear supernatant was filtered at 0.2 μm (Acrodisc syringe filters with GHP membranes) and kept in clean tubes at -20°C until processing. HPLC analysis was carried out according to the method described by Adom et al. (2003) with modifications. Samples were manually shaken, then 200 μL was extracted and placed in vials for HPLC autosampling. The mobile phase was 0.25% (v/v) trifluoroacetic acid (TFA, solvent A) and pure ACN (solvent B). The HPLC gradient was linear: after 2 μL sample injection, solvent B was kept at 4% for 1.16 min, then increased gradually to 12% over 1.16 min, to 23% over 4.63 min, to 95% over 1.85 min, and to 100% over 1.16 min, with final rate maintained for a further 2.78 min. The duration of the analysis was 11.58 min at a solvent flow rate of 1.1 mL min^-1^. The HPLC equipment (Shimadzu, Kyoto, Japan) had a UV diode array detector (SPD-M20A) at wavelength 282 nm, and an Ultra Tech sphere C18 analytical column (33 mm × 4.6 mm i.d., 1.5 μm particle size; Cil Cluzeau, Sainte-Foy-La-Grande, France) kept at 36°C. Control sample solutions of shoots containing known phenolic acid concentrations were analyzed at the beginning of each new batch analysis, and measurement accuracy was verified by checking expected concentrations.

Each peak was identified by analyzing the retention time and absorbance spectrum of each pure compound (i.e., *p-*Coumaric, caffeic, syringic, vanillic and *t*-ferulic acids; Supplementary Figure S1). The coefficients of determination of all calibration curves were >99%.

### Quantitative Determination of Sugars

Shoots (5 g) were homogenized in methanol (20 mL) with an Ultra Turrax T25 at 13,500 rpm for 30 s until they attained uniform consistency. Samples were filtered once through filter paper (589 Schleicher), and a second time through cellulose acetate syringe filters (0.45 μm). The extract was then ready for HPLC analysis, for which we used a Jasco X-LC liquid chromatography system (Jasco Inc., Easton, MD, United States) consisting of a PU-2080 pump, an MD-2015 multiwavelength detector, an AS-2055 autosampler, and a CO-2060 column oven interfaced to a PC using the ChromNAV chromatography data system software (Jasco Inc., Easton, MD, United States).

Sugars were separated in a HyperRez XP Carbohydrate Pbþþ analytical column (7.7 mm × 300 mm; ThermoFisher Scientific, Waltham, MA, United States), operating at 80°C. Isocratic elution was carried out with water at a flow rate of 0.6 mL min^-1^. D-(β)-glucose and D-(β)- fructose were quantified by a calibration method. Standards were dissolved in water and the calibration curves were generated with concentrations ranging from 100 mg L^-1^ to 1,000 mg L^-1^ (Nicoletto et al., 2013).

### Statistical Analysis

The data are the means of measurements from three different pots per treatment. The analysis of variance (ANOVA) was performed in the SPSS 23 (IBM Corp) software, and was followed by pairwise *post hoc* analyses (Student–Newman–Keuls test) to determine significant differences among means at *P* ≤ 0.05.

## Results

### Audus Test and Effects of Sedaxane on Shoot and Root Growth

**FIGURE 1:**
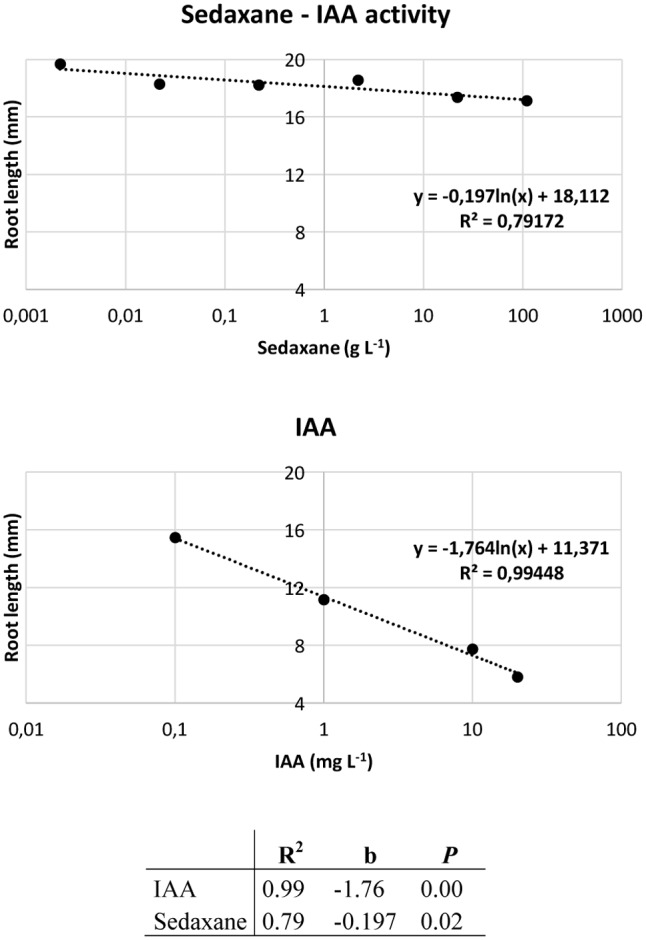
Audus test: auxin-like activity of sedaxane measured as variations in root length in watercress. Linear regression analysis (below) performed on 20 samples and averaged over 5 replicates. Note that the x axis has a logarithmic scale.

**FIGURE 2:**
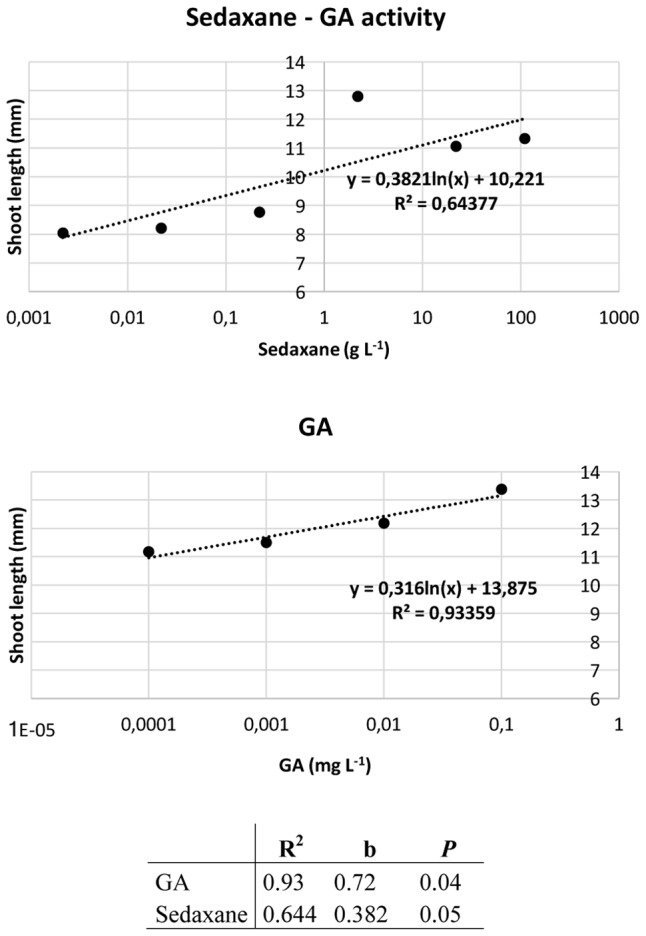
Audus test: gibberellin-like activity of sedaxane measured as variations in shoot length in lettuce. Linear regression analysis (below) was performed on 20 samples and averaged over five replicates. Note that the *x*-axis has a logarithmic scale.

Ahead of the analysis, the Audus test was used to determine the biostimulant properties of the active ingredient sedaxane. As with the natural auxin IAA, which reduces root elongation in the model plant watercress and is dose-proportional, increasing concentrations of sedaxane led to a progressive reduction in root length, suggesting an auxin-like effect (**Figure 1**). We also found sedaxane to exhibit gibberellin-like activity, as it enhanced the shoot growth of lettuce and had a similar dose-proportionality to exogenous gibberellic acid (**Figure 2**). Both regression curves were significant (*P* < 0.02 for root responses, *P* ≤ 0.05 for shoot responses), revealing the hormone-like activity of this fungicide.

Under sterile conditions, fungicide treatment did not significantly enhance plant growth, although the medium dose of sedaxane (75 μg seed^-1^) appreciably increased shoot (+21%) and root (+10%) biomasses as compared to untreated controls (**Table 1**). The effects of the seed treatments were more evident on other root features: Root length increased by 60% and root area by 45% at the intermediate fungicide dose. While root diameter was slightly smaller (*P* > 0.05), the number of root tips and forks increased, most noticeably with the intermediate (tips +27%, forks +51%) and maximum doses (tips +17%, forks +48%), although only the root branching increase was significant. These results show that root stimulation by sedaxane may be dose-dependent up to saturation.

**Table 1:** Main shoot and root parameters (mean ± SE; *n* = 3) in *Zea mays* at 20 days after sowing (DAS) in sterilized pot soil under increasing seed-applied doses of sedaxane.

Sedaxane dose (μg seed^-1^)	Shoot	Root					
		
	DW (g plant^-1^)	DW (g plant^-1^)	Length (m plant^-1^)	Area (m^2^ plant^-1^)	Diameter (mm)	Tips (n plant^-1^)	Forks (n plant^-1^)
0	0.50 ± 0.07^a^	0.24 ± 0.03^a^	130.2 ± 35^b^	0.23 ± 0.04^b^	1.81 ± 0.13^a^	6657 ± 1769^a^	10594 ± 2280^a^
25	0.52 ± 0.04^a^ (+3)	0.25 ± 0.03^a^ (+3)	167.4 ± 6^ab^ (+29)	0.26 ± 0.01^ab^ (+14)	1.54 ± 0.03^a^ (-15)	6180 ± 957^a^ (-7)	12649 ± 1681^ab^ (+19)
75	0.61 ± 0.04^a^ (+21)	0.26 ± 0.01^a^ (+10)	208.0 ± 24^a^ (+60)	0.33 ± 0.03^a^ (+45)	1.59 ± 0.10^a^ (-12)	7784 ± 994^a^ (+17)	15985 ± 1849^b^ (+51)
150	0.53 ± 0.04^a^ (+6)	0.24 ± 0.01^a^ (+1)	184.9 ± 16^ab^ (+42)	0.31 ± 0.02^ab^ (+37)	1.68 ± 0.10^a^ (-7)	8467 ± 405^a^ (+27)	15711 ± 718^b^ (+48)


**Letters indicate significant differences among treatments within the same parameter (Student–Newman–Keuls test, *P* ≤ 0.05). In brackets: % variation *vs*. untreated controls.**

### Effects of Sedaxane on SPAD, Protein and Sugar Contents

Leaf greenness, measured in terms of SPAD values, was very stable across treatments at the end of the trial (**Table 2**), while protein content was significantly influenced by sedaxane (*P* < 0.001), with an increase of 14% at the intermediate and highest AI doses (**Table 2**). A similar effect was found in the roots, with protein content increasing significantly at the highest AI dose (+20% *vs*. untreated controls).

**Table 2:** Leaf SPAD values, shoot and root protein, glucose, and fructose contents (mean ± SE; *n* = 9) in *Zea mays* at 20 days after sowing (DAS) in sterilized pot soil under increasing seed-applied doses of sedaxane.

Sedaxane dose (μg seed^-1^)	Shoot				Root		
		
	SPAD	Protein (mg g^-1^ FW)	Glucose (μg g^-1^ FW)	Fructose (μg g^-1^ FW)	Protein (mg g^-1^ FW)	Glucose (μg g^-1^ FW)	Fructose (μg g^-1^ FW)
0	34.6 ± 0.8^a^	5.6 ± 0.1^b^	3328 ± 170^a^	1005 ± 24^a^	1.5 ± 0^b^	3819 ± 128^a^	1322 ± 56^a^
25	34.1 ± 0.2^a^ (-1)	5.9 ± 0.1^ab^ (+5)	3310 ± 33^a^ (-1)	792 ± 35^b^ (-21)	1.5 ± 0.1^b^	3724 ± 163^a^ (-2)	1418 ± 109^a^ (+7)
75	34.7 ± 0.9^a^ (+0.5)	6.4 ± 0.2^a^ (+14)	3340 ± 113^a^	1012 ± 59^a^ (+1)	1.5 ± 0.1^b^	3665 ± 140^a^ (-4)	1476 ± 125^a^ (+12)
150	34.3 ± 0.8^a^ (-0.5)	6.4 ± 0.2^a^ (+14)	3518 ± 70^a^ (+6)	855 ± 14^b^ (-15)	1.8 ± 0^a^ (+20)	3859 ± 384^a^ (+1)	1313 ± 191^a^ (-1)


**Letters indicate significant differences among treatments within the same parameter (Student–Newman–Keuls test, *P* ≤ 0.05). In brackets: % variation *vs*. untreated controls.**

Fungicide treatment did not affect the shoot and root glucose content, the former having an average concentration of 3374 μg g^-1^ FW, the latter 3766 μg g^-1^ FW. The only variation found with regard to fructose was that it was significantly reduced in the shoot at the lowest and highest sedaxane doses (-21% and -15%, respectively, *vs*. untreated controls) (**Table 2**).

### Variations in GS and GOGAT Activities with Sedaxane

**FIGURE 3:**
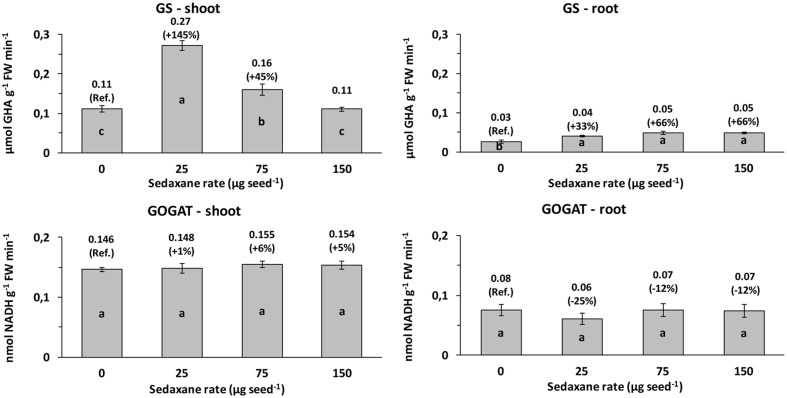
Shoot and root glutamine synthetase (GS) and glutamate synthase (GOGAT) activities (mean ± SE; *n* = 9) in *Zea mays* at 20 days after sowing (DAS) in sterilized pot soil under increasing seed-applied doses of sedaxane. Letters indicate significant differences among treatments within the same parameter (Student–Newman–Keuls test, *P* ≤ 0.05). In brackets: % variation *vs*. untreated controls.

Glutamine synthetase (GS) activity and glutamate synthase (GOGAT) activity were, respectively, 3.8 and 2.1 times higher, on average, in the shoots than in the roots. Seed treatment with sedaxane significantly increased GS activity in the shoots (*P* < 0.01) at the lowest (+145% *vs*. controls) and intermediate AI doses (+45%), and in the roots (*P* < 0.001), particularly at the intermediate and highest AI doses (both +66%, *P* ≤ 0.05) (**Figure 3**).

Sedaxane treatments did not affect GOGAT activity in the shoots, while slight, but insignificant, reductions were observed in the roots (**Figure 3**).

### Effect of Sedaxane on Leaf Phenylpropanoid Metabolism

A significant increase in soluble phenolic acids in the shoots was observed at the lowest sedaxane dose (+14% *vs*. untreated controls), while values similar to controls were detected at greater AI doses (*P* ≤ 0.05) (**Table 3**). However, when individual compounds were analyzed, large differences among treatments were detected for caffeic acid, and, to a lesser extent, for syringic and *p-*coumaric acids (*P* ≤ 0.05). Significantly higher concentrations of caffeic acid were found in all treated plants compared with untreated controls (*P* ≤ 0.05). Sedaxane increased caffeic acid by 41–58%, depending on the dose, and *p-*coumaric acid, the most abundant phenolic compound, by 23% at the lowest and 19% at the intermediate dose. There were only slight differences in the vanillic and *t*-ferulic acid contents in treated plants as compared with controls (*P* > 0.05).

**Table 3:** Shoot phenylalanine ammonia-lyase activity (PAL), soluble phenol content and phenolic acid profiles (mean ± SE; *n* = 9) of *Zea mays* at 20 days after sowing (DAS) in sterilized pot soil under increasing seed-applied doses of sedaxane.

Sedaxane dose (μg seed^-1^)	PAL (nmol cinn. acid mg^-1^ prot. min^-1^)	Soluble phenols (as mg gallic acid g^-1^ FW)	Vanillic acid (μg g^-1^ FW)	Caffeic acid (μg g^-1^ FW)	Syringic acid (μg g^-1^ FW)	*p*-coumaric acid (μg g^-1^ FW)	*t*-ferulic acid (μg g^-1^ FW)
0	3.1 ± 0.12^b^	36.4 ± 1.5^b^	0.78 ± 0.06^a^	2.88 ± 0.24^b^	11.7 ± 0.6^b^	21 ± 0.9^b^	0.72 ± 0.04
25	3.99 ± 0.28^a^ (+29)	41.4 ± 0.8^a^ (+14)	0.63 ± 0.06^a^ (-19)	4.14 ± 0.24^a^ (+44)	14.6 ± 0.9^a^ (+25)	25.8 ± 1.9^a^ (+23)	0.83 ± 0.07 (+15)
75	3.08 ± 0.22^b^ (-1)	35.1 ± 1^b^ (-4)	0.63 ± 0.03^a^ (-19)	4.55 ± 0.43^a^ (+58)	13.8 ± 0.6^ab^ (+18)	24.9 ± 0.6^a^ (+19)	0.82 ± 0.03 (+14)
150	4.42 ± 0.26^a^ (+43)	35.2 ± 2^b^ (-3)	0.63 ± 0.05^a^ (-19)	4.06 ± 0.34^a^ (+41)	12.4 ± 0.6^ab^ (+6)	21.9 ± 1^b^ (+4)	0.74 ± 0.03 (+3)


**Letters indicate significant differences among treatments within the same parameter (Student–Newman–Keuls test, *P* ≤ 0.05). In brackets: % variation *vs*. untreated controls.**

The ANOVA revealed a significant increase (*P* ≤ 0.05) in PAL enzyme activity in the shoots with the lowest and highest fungicide doses (+29% and +43%, respectively) as compared with untreated controls (**Table 3**).

## Discussion

Sedaxane belongs to the new class of succinate dehydrogenase inhibitors, and is currently used as a seed-coating fungicide on various crops in several countries, with registration approval being increasingly granted worldwide. It is a broad-spectrum antifungal agent, and is of particular interest in combatting *Rhizoctonia solani* and *Mycosphaerella reliana* in maize.

In light of previous results on root stimulation in wheat (Barchietto et al., 2012), we investigated the side-effects of sedaxane in maize over and above its protective capacity, and found that seed treatment significantly modified morphological traits and physiological activities in disease-free plants grown in sterile soil.

The Audus test is considered to be the most reliable bioassay in terms of reproducibility and repeatability for verifying and quantifying the biostimulant activity of molecules in plants, and can be used to ascertain whether an exogenous compound has auxin- and/or gibberellin-like activity (Conselvan et al., 2017). Auxin (IAA) is the most important hormone in plants, and is involved in several plant growth and development phases, such as embryogenesis, organogenesis, tissue patterning and tropisms (Davies, 2010). Molecular genetic studies have brought to light the central role of auxin in primary root elongation, lateral root initiation, and root hair development (De Smet et al., 2006; Overvoorde et al., 2010). The phytohormone gibberellin (GA) also modulates plant development by lengthening roots and stems, and expanding leaves (Fleet and Sun, 2005). We used an Audus bioassay to demonstrate that sedaxane has both auxin- and gibberellin-like activity, as the confirmation of its biostimulant properties.

Although the improvements in aerial and root biomasses detected in this trial were not significant, we found that root length and area, and the number of root tips and branches increased almost in proportion to the dose of sedaxane, consistent with results reported by Colla et al. (2014) on maize coleoptile elongation with protein hydrolysates. All these root morphology modifications are known responses to biostimulant compounds (Calvo et al., 2014). Root development is essential for plant survival as it plays a crucial role in water and nutrient acquisition for growth, the synthesis and accumulation of secondary metabolites, and interaction with soil organisms (Saini et al., 2013).

The data collected from this trial are consistent with Barchietto et al. (2012) regarding stimulation of wheat shoots and roots by seed-applied sedaxane. At 30 days after sowing (DAS), they observed significant increases in root length in treated plants as compared with controls, and no differences in root biomass, as in our case study at 20 DAS. Interestingly, they also found that at 60 DAS root length was unaffected by sedaxane seed treatment, whereas root biomass increased significantly (+39–87%, according to variety).

In the sterile soil conditions of our trial, the SPAD value was very stable across treatments, but this was not the case in the supplementary trial we carried out in unsterile soil conditions to investigate the potential effect of sedaxane in field-like conditions, where we found a slight but significant increase in SPAD (up to 7%) (Supplementary Table S1). This result is in line with practical expectations in the field given the correlation between SPAD and photosynthetic activity, the N status of the plant and protein contents (Prost and Jeuffroy, 2007; Sim et al., 2015).

It should be noted that sedaxane may affect not only fungal mitochondria but also the SDH complex II of plants, partially inhibiting its activity (Avenot and Michailides, 2010). Fuentes et al. (2011) reported better photosynthetic performance in *Arabidopsis* plants with compromised expression of the flavoprotein subunit of SDH than in wild-type plants. Inhibition of the SDH subunit also resulted in an increase in the number and aperture of leaf stomata, which significantly increased CO_2_ assimilation, in turn enhancing growth and protein production. Araújo et al. (2011) obtained similar results with tomato plants with antisense inhibition of the iron-sulfur subunit of SDH. However, the higher SPAD values of sedaxane-treated maize observed in our supplementary study with unsterilized soil may also be related to a slowing down of chlorophyll molecule degradation, as reported for fungicides of the Strobilurin class (Grossmann and Retzlaff, 1997; Xu and Huang, 2009). However, this hypothesis needs to be confirmed by studying SDH activity and chlorophyll content in sedaxane-treated plants.

The higher protein content in sedaxane-treated seedlings may be ascribed to better nitrogen metabolism through the activity of the enzymes involved. In fact, the GS/GOGAT metabolic pathway is the main route of N assimilation in higher plants (Mokhele et al., 2012), allowing ammonium taken up directly or originating from nitrate to be assimilated into amino acids (Xu et al., 2012). The GS enzyme is also critical for re-assimilation of the NH_4_^+^ constantly released in large amounts via photorespiration, phenylalanine consumption for lignin biosynthesis, and protein catabolism (Lea and Miflin, 2011). GS activity, which increased significantly following sedaxane application, therefore plays a pivotal role in many aspects of plant development (Seabra and Carvalho, 2014), as it is a key component in nitrogen use efficiency (NUE) and plant yield (Thomsen et al., 2014).

GS and GOGAT enzyme activities have been previously reported to be affected by biostimulants (Baglieri et al., 2014). Our data are consistent with those of Ajigboye et al. (2016), who found that improvements in the photosynthetic efficiency, growth, and biomass of sedaxane-treated wheat plants were associated with up- or down-regulation changes in gene expression, and consequent modifications of physiological processes, particularly under drought stress conditions. In particular, sedaxane is reported to induce transcriptional regulation of genes and transcriptional factors, altering the flavonoid and phenolic metabolism (Ajigboye et al., 2016). Our study confirmed that sedaxane stimulates phenylpropanoid metabolism in maize as we found an increase in PAL enzyme activity, although, unexpectedly, the effect was not observed at the intermediate dose. The PAL enzyme catalyzes the first metabolic step from primary to secondary metabolism (Douglas, 1996), deaminating phenylalanine to produce cinnamic acid. As a consequence, there was an increase in the total content of phenolic compounds in shoot tissues from seedlings treated with the lowest concentration of sedaxane, but not at the highest dose. However, there were substantial changes in the concentrations of individual phenolic acids in relation to fungicide application: In particular, there was a considerable increase in caffeic acid in treated plants, which may be of interest in view of its weak auxin-like effect (Lavee et al., 1986; Ishikura et al., 2001; Nagasawa et al., 2016). The main precursor of lignin in the cell wall of gramineous plants is *p*-coumaric acid, and a greater abundance of it in sedaxane-treated plants could contribute to more intense cell activity and division. Vanillic and *p*-coumaric acids are also reported to be antifungal phenols, meaning that sedaxane may also contribute indirectly to plant defense (Lattanzio et al., 2006; Zabka and Pavela, 2013; Pusztahelyi et al., 2015). Stimulation of the secondary metabolism may also be explained by enhanced primary metabolism activity, as evidenced by the protein and sugar contents (**Table 2**).

As with other SDHIs studied in wheat, all the physiological changes brought about by sedaxane may also delay senescence, and improve the yield and protein content of maize plants (Bayles, 1999; Dimmock and Gooding, 2002; Zhang et al., 2010; Abdelrahman et al., 2017), but this requires further investigation in current field conditions.

## Conclusion

Sedaxane has a considerable effect on rooting power of maize, particularly on the length, surface area and number of lateral roots. This study found that sedaxane exhibits biostimulant activity in maize seedlings due to its hormone-like activities, corroborated by the fact that most of the observed effects are saturated at moderate doses, as with phytohormones. We have high expectations that seed treatment with this fungicide will facilitate plant establishment, and may provide particular benefits under adverse soil and climatic conditions. Stimulation of the enzyme activities involved in N assimilation and phenylpropanoid metabolism is in agreement with previous findings on this active ingredient and other SDHI fungicides, and is consistent with improved N status and antioxidant activity.

As the fungicide doses tested here are within the recommended label range, the biostimulant activity of sedaxane is an additional benefit, over and above its protective role against seed- and soil-borne diseases, which could be exploited in the cultivation of maize. Although further studies are needed to see whether these improvements also influence final growth and yield, our preliminary results suggest that, as things currently stand, roots may be enhanced in the early growth stages, even in non-sterile soil.

## Author Contributions

CDC oversaw the greenhouse trial, assisted with the laboratory analyses, collected and analyzed the data, and wrote the first draft of the manuscript. GBC performed all the enzymatic and biological assays, and also collected and analyzed the data, carried out the literature research and improved the manuscript content. GB performed the HPLC analysis and assisted with analysis of the statistical data. PC helped design the experiment, analyzed the data and improved the manuscript content. LS helped revise the text. TV conceived the research idea, and corrected and arranged the final version of this work. All authors contributed to the interpretation and discussion of the results.

## Conflict of Interest Statement

The research was funded by Syngenta Crop Protection (Basel, Switzerland), which also provided the seeds and fungicide. Collection of data, analysis and interpretation, as well as article writing was carried out by the authors independently. The authors have the full data set available. The authors declare that the research was conducted in the absence of any commercial or financial relationships that could be construed as a potential conflict of interest.
