# Supplementary material for: Biostimulant Effects of Seed-Applied Sedaxane Fungicide: Morphological and Physiological Changes in Maize Seedlings
Source: Front Plant Sci. 2017 Dec 6;8:2072. doi: 10.3389/fpls.2017.02072 (PMC5723653; doi:10.3389/fpls.2017.02072)
Supplement: Supplementary file 1 [file Table_1.docx]

**Supplementary Table 1**. Main shoot and root parameters (mean ± se; n = 3) in *Zea mays* at 20 days after sowing (DAS) in unsterilised pot soil at increasing seed-applied doses of sedaxane. Letters indicate significant differences among treatments within same parameter (Student-Newman-Keuls test, *P* ≤ 0.05). In brackets: % variation *vs*. untreated controls.

| **Sedaxane rate**  **(μg seed^-1^)** | **Shoot** | | **Root** | | | | | |
| --- | --- | --- | --- | --- | --- | --- | --- | --- |
|  | **DW**  **(g plant^-1^)** | **SPAD** | **DW**  **(g plant^-1^)** | **Length**  **(m plant^-1^)** | **Area**  **(m^2^ plant^-1^)** | **Diameter**  **(mm)** | **Tips**  **(n plant^-1^)** | **Forks**  **(n plant^-1^)** |
| 0 | 0.35±0.03**^a^** | 27.9±0.7**^b^** | 0.17±0.01**^a^** | 137.4±9**^b^** | 0.21±0.01**^b^** | 1.55±0.05**^a^** | 4285±215**^b^** | 9798±1010**^a^** |
| 25 | 0.34±0.05**^a^** (-3) | 28.4±0.6**^ab^** (+2) | 0.17±0.01**^a^** | 155.5±21**^ab^** (+13) | 0.23±0.03**^ab^** (+10) | 1.49±0.01**^ab^** (-4) | 3804±618**^b^** (-11) | 10111±1277**^a^** (+3) |
| 75 | 0.42±0.02**^a^** (+20) | 28.4±0.7**^ab^** (+2) | 0.19±0.005**^a^** (+12) | 156.6±11**^ab^** (+14) | 0.23±0.02**^ab^** (+10) | 1.50±0.04**^ab^** (-3) | 4190±399**^b^** (-2) | 10298±796**^a^** (+5) |
| 150 | 0.42±0.04**^a^** (+20) | 29.9±0.6**^a^** (+7) | 0.20±0.01**^a^** (+18) | 189.1±19**^a^** (+38) | 0.27±0.02**^a^** (+29) | 1.43±0.03**^b^** (-8) | 5479±131**^a^** (+28) | 11628±958**^a^** (+19) |
