# Supplementary material for: Biostimulant Effects of Seed-Applied Sedaxane Fungicide: Morphological and Physiological Changes in Maize Seedlings
Source: Front Plant Sci. 2017 Dec 6;8:2072. doi: 10.3389/fpls.2017.02072 (PMC5723653; doi:10.3389/fpls.2017.02072)
Supplement: Supplementary file 2 [file Image_1.pdf]

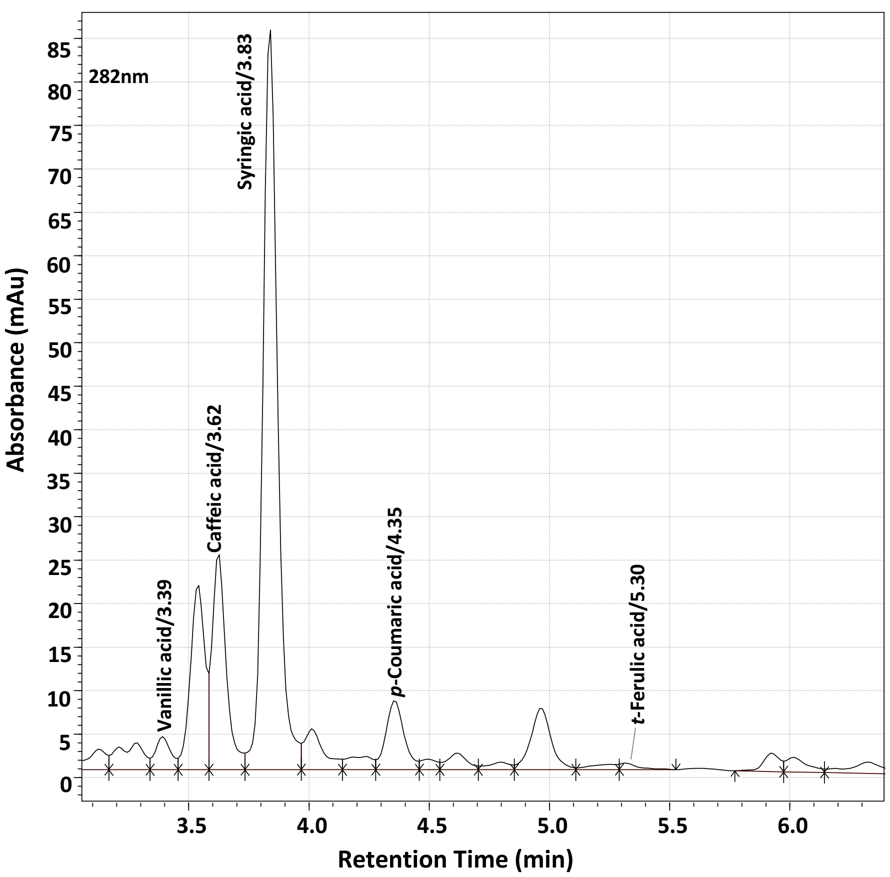

Supplementary Figure 1. Sample chromatogram of phenolic aid HPLC determination. The mobile phase was 0.25% (v/v) trifluoroacetic acid (TFA, solvent A) and pure ACN (solvent B). The HPLC gradient was linear: after 2 µL sample injection, solvent B was kept at 4% for 1.16 min, then increased gradually to 12% in 1.16 min, to 23% in 4.63 min, to 95% in 1.85 min, to 100% in 1.16 min, and the final rate was maintained for a further 2.78 min. Analysis had a duration of 11.58 min at a solvent flow rate of 1.1 mL min^{-1}. The HPLC equipment (Shimadzu, Kyoto, Japan) had a UV diode array detector (SPD-M20A) at wavelength 282 nm, and an Ultra Tech sphere C18 analytical column (33 mm × 4.6 mm i.d., 1.5 µm particle size; Cil Cluzeau, Sainte-Foy-La-Grande, France) kept at 36 ° .
